# The Aging Stomach: Clinical Implications of *H. pylori* Infection in Older Adults—Challenges and Strategies for Improved Management

**DOI:** 10.3390/ijms252312826

**Published:** 2024-11-28

**Authors:** Jaroslaw Skokowski, Yogesh Vashist, Sergii Girnyi, Tomasz Cwalinski, Piotr Mocarski, Carmine Antropoli, Antonio Brillantino, Virginia Boccardi, Aman Goyal, Francesco A. Ciarleglio, Muhannad Abdullah Almohaimeed, Raffaele De Luca, Adel Abou-Mrad, Luigi Marano, Rodolfo J. Oviedo, Beata Januszko-Giergielewicz

**Affiliations:** 1 Department of Medicine, Academy of Applied Medical and Social Sciences-AMiSNS: Akademia Medycznych I Spolecznych Nauk Stosowanych, 82-330 Elbląg, Poland; b.januszko-giergielewicz@amisns.edu.pl; 2 Department of General Surgery and Surgical Oncology, “Saint Wojciech” Hospital, “Nicolaus Copernicus” Health Center, 80-000 Gdańsk, Poland; sgirnyi@copernicus.gda.pl (S.G.); tcwalinski@copernicus.gda.pl (T.C.); pmocarski@copernicus.gda.pl (P.M.); 3 Organ Transplant Center for Excellence, Center for Liver Diseases and Oncology, King Faisal Specialist Hospital and Research Center, 12211 Riyadh, Saudi Arabia; y.vashist@kfu.edu.sa (Y.V.); malmehaimad@kfshrc.edu.sa (M.A.A.); 4 Department of Surgery, Antonio Cardarelli Hospital, 80100 Naples, Italy; carmine.antropoli@aocardarelli.it (C.A.); antonio.brillantino@aocardarelli.it (A.B.); 5 Division of Gerontology and Geriatrics, Department of Medicine and Surgery, University of Perugia, 06132 Perugia, Italy; virginia.boccardi@unipg.it; 6 Adesh Institute of Medical Sciences and Research, 151001 Bathinda, Punjab, India; doc.aman.goyal@gmail.com; 7 Department of General Surgery and Hepato-Pancreato-Biliary (HPB) Unit-APSS, 38121Trento, Italy; francesco.ciarleglio@apss.tn.it; 8 Department of Surgical Oncology, IRCCS Istituto Tumori “Giovanni Paolo II”, 70100 Bari, Italy; raffaele.deluca@oncologico.bari.it; 9 Department of Surgery, Centre Hospitalier Universitaire d’Orléans, 45100 Orléans, France; adel.abou-mrad@orange.fr; 10 Department of Medicine, Surgery, and Neurosciences, University of Siena, 53100 Siena, Italy; 11 Department of Surgery, Nacogdoches Medical Center, Nacogdoches, TX 75965, USA; roviedo3@central.uh.edu; 12 Department of Surgery, University of Houston Tilman J. Fertitta Family College of Medicine, Houston, TX 75961, USA; 13 Department of Surgery, Sam Houston State University College of Osteopathic Medicine, Conroe, TX 77301, USA

**Keywords:** aging, helicobacter pylori, immunosenescence, geriatric gastroenterology, probiotic therapy

## Abstract

Aging is a multifactorial biological process characterized by a decline in physiological function and increasing susceptibility to various diseases, including malignancies and gastrointestinal disorders. Helicobacter pylori (*H. pylori*) infection is highly prevalent among older adults, particularly those in institutionalized settings, contributing to conditions such as atrophic gastritis, peptic ulcer disease, and gastric carcinoma. This review examines the intricate interplay between aging, gastrointestinal changes, and *H. pylori* pathogenesis. The age-associated decline in immune function, known as immunosenescence, exacerbates the challenges of managing *H. pylori* infection. Comorbidities and polypharmacy further increase the risk of adverse outcomes in older adults. Current clinical guidelines inadequately address the specific needs of the geriatric population, who are disproportionately affected by antibiotic resistance, heightened side effects, and diagnostic complexities. This review focuses on recent advancements in understanding *H. pylori* infection among older adults, including epidemiology, diagnostics, therapeutic strategies, and age-related gastric changes. Diagnostic approaches must consider the physiological changes that accompany aging, and treatment regimens need to be carefully tailored to balance efficacy and tolerability. Emerging strategies, such as novel eradication regimens and adjunctive probiotic therapies, show promise for improving treatment outcomes. However, significant knowledge gaps persist regarding the impact of aging on *H. pylori* pathogenesis and treatment efficacy. A multidisciplinary approach involving gastroenterologists, geriatricians, and other specialists is crucial to providing comprehensive care for this vulnerable population. Future research should focus on refining diagnostic and therapeutic protocols to bridge these gaps, ultimately enhancing clinical outcomes and reducing the burden of *H. pylori*-associated diseases in the aging population.

## 1. Introduction

Aging is an intricate biological process that affects individuals across molecular, cellular, tissue, and systemic levels. It is characterized by a gradual decline in the body’s ability to repair its cells, which in turn increases susceptibility to diseases typically associated with aging, such as cancer. As populations worldwide, particularly in countries like Japan, Italy, and the United States [1,2,3], continue to grow older, the health challenges arising from this demographic shift become increasingly significant. Moreover, nearly 40% of people over 60 years old experience gastrointestinal issues, a consequence often linked to the natural decline in digestive function that accompanies aging [4]. Older individuals are particularly prone to upper gastrointestinal disorders, which manifest as atrophic changes in the gastric mucosa, reduced activity of digestive enzymes, and an increased risk of infections, with *Helicobacter pylori* (*H. pylori*) being particularly notable [5,6,7]. Chronic infection with *H. pylori* in older patients is a well-established precursor to conditions such as atrophic gastritis, intestinal metaplasia, and gastric cancer [8,9,10,11]. Although *H. pylori* infection has a significant impact on this demographic group, current clinical guidelines often fail to fully address the specific risks, treatment options, and potential side effects for older patients [12,13,14,15]. Additionally, aging is accompanied by a process known as immunosenescence, a gradual decline in immune function that can exacerbate the severity of diseases, especially in individuals who are malnourished or otherwise at risk [16,17,18]. This decline in immune efficacy complicates the management of *H. pylori* infections in older adults, presenting a significant challenge in balancing effective treatment while minimizing adverse effects [8,19].

Although modern healthcare advancements have extended life expectancy, the aging population is now confronted with a higher prevalence of chronic diseases and prolonged periods of disability [20,21,22,23,24]. The complex interaction between *H. pylori* infection and the aging process presents significant clinical challenges that are not yet fully addressed in the literature. While research has extensively explored the role of *H. pylori* in the development of gastric cancer [25], there remains a significant gap in understanding how aging influences these pathological processes [26]. The unique difficulties in diagnosing and treating *H. pylori* in older patients, who often present with multiple comorbidities and compromised immune systems, are underrepresented in current studies [27,28].

This narrative review aims to fill these gaps by compiling recent research on *H. pylori* infection in older patients, with an emphasis on epidemiology, diagnostic challenges, therapeutic strategies, and the potential adverse events associated with treatment. The ultimate goal is to provide insights that can improve clinical practice and lead to better outcomes for this increasingly vulnerable segment of the population.

## 2. Methodology

A comprehensive literature search was conducted across PubMed, Medline, and Cochrane databases from the earliest available literature to 3 September 2024. The search aimed to identify all relevant articles published using medical subject heading keywords such as “aging”, “gastric cancer”, “older persons”, “Helicobacter pylori”, “inflammation”, and “immunosenescence”. These keywords were employed in various combinations to ensure a thorough retrieval of pertinent studies, encompassing research from basic science, animal models, and clinical trials. In this review, “older adults” are defined as individuals aged 60 years and older, in line with the WHO classification and the age group commonly targeted in studies involving age-related changes in gastrointestinal function [29].

Inclusion criteria required studies to focus on individuals aged 60 years and older, specifically addressing the diagnosis, management, or treatment of *H. pylori* infections in this population. Both qualitative and quantitative research, including observational studies, randomized controlled trials (RCTs), systematic reviews, and meta-analyses, were deemed eligible. Due to resource constraints, only articles published in English were reviewed. Priority was assigned to studies that provided substantial contributions to the understanding of epidemiology, diagnostic approaches, therapeutic interventions, and complications associated with *H. pylori* among the elderly. The exclusion criteria encompassed studies that involved populations younger than 60 years or those lacking explicit age-related data. Articles that did not address core topics such as *H. pylori* infection, aging, immunosenescence, or gastric cancer in older adults were excluded. Additionally, case reports, editorials, conference abstracts, and letters that did not present substantial empirical data were omitted. In instances of duplicate publications, the most comprehensive version of the study was retained. Basic science studies, including animal and in vitro research, were reviewed for their contextual relevance; however, only those demonstrating clear clinical implications for older adults were included in the final analysis.

The methodological rigor applied in the selection process ensured that the review focused on studies with a high level of relevance and applicability to clinical practice, thereby providing an evidence-based synthesis of current knowledge on *H. pylori* infection in the aging population.

## 3. Patterns and Determinants of *H. pylori* Infection in Older Populations

### 3.1. Epidemiology

**Figure 1:**
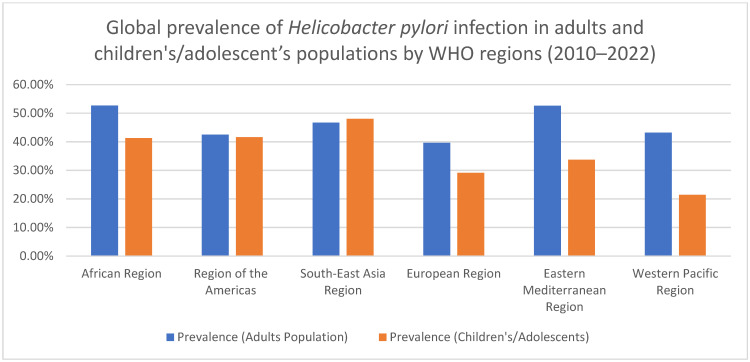
Figure: global prevalence of Helicobacter pylori infection in adults and children/adolescent populations by WHO regions (2010–2022).

The epidemiology of *H. pylori* infection in older patients reveals a complex and varied picture. Although improvements in healthcare have led to a decline in *H. pylori* prevalence among younger and middle-aged groups, the infection remains common among older adults, and its associated complications continue to increase with age [30]. The prevalence of *H. pylori* infection varies significantly depending on factors such as socioeconomic status, geographic location, and the specific populations studied. In developing countries, higher prevalence rates are observed in children, largely due to lower socioeconomic conditions, poor hygiene, and overpopulation [31]. Conversely, in developed countries, prevalence tends to increase with age, likely reflecting the exposure of older generations to poorer sanitation conditions in the past [30] (Figure 1).

In older persons, particularly those in institutionalized settings, *H. pylori* infection rates are alarmingly high, ranging from 70% to 85% [32,33]. Some studies have even reported rates as high as 86.5% among hospitalized older patients, many of whom are residents of nursing homes [34]. However, there is a marked reduction in prevalence among those over 85 years, possibly due to chronic atrophic gastritis (CGA) and the extensive use of antibiotics and antisecretory drugs [34,35].

Overall, *H. pylori* prevalence in older patients is generally high, especially among those with gastrointestinal diseases, where rates exceed 70%, and among asymptomatic individuals, with approximately 60% affected [4,35,36]. Although many older patients do not exhibit symptoms, a significant proportion—between 10% and 20%—will develop peptic ulcer disease (PUD), and around 1% may progress to more serious conditions such as gastric cancer or mucosa-associated lymphoid tissue (MALT) lymphoma [37,38,39,40].

Older adults face a higher risk of severe complications from *H. pylori* infection, leading to increased rates of hospitalization and mortality compared to younger populations [41,42]. This vulnerability is due to several factors, including the often subtle or atypical presentation of *H. pylori* in older patients, which can delay diagnosis [43]. Additionally, the presence of multiple comorbidities and the use of medications that damage the gastric mucosa, such as non-steroidal anti-inflammatory drugs (NSAIDs), further increase the risks associated with *H. pylori* [41]. In fact, *H. pylori* and NSAID use are significant independent risk factors for PUDs, with co-infected NSAID users facing dramatically increased ulcer risk [44,45]. The combined impact of *H. pylori* and NSAIDs is particularly harmful in older patients, as both factors compromise the gastric mucosal defenses. Age-related decreases in protective prostaglandin secretion and gastric acid production, often linked to fundal atrophic gastritis, further weaken these defenses [36]. Moreover, antibiotic resistance, particularly to clarithromycin and levofloxacin, is more prevalent in older patients, complicating treatment and underscoring the need for precise diagnosis and effective management of *H. pylori* infection in this vulnerable population [13,23,46].

### 3.2. Gastric Aging: Functional Decline, Microbial Shifts, and Increased Disease Risk

**Figure 2:**
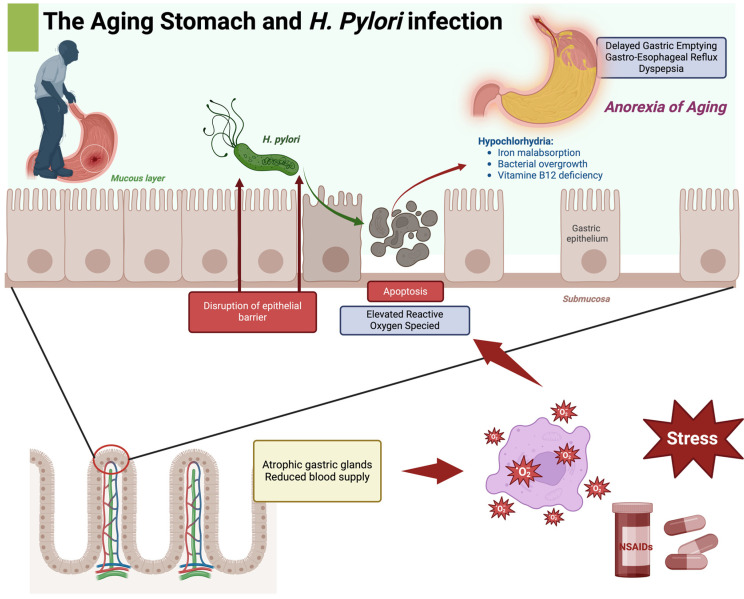
This image illustrates the impact of *H. pylori* infection on the aging stomach and its potential role in “Anorexia of Aging”. The image shows how *H. pylori* can penetrate the mucus layer of the stomach, causing disruption of the epithelial barrier and apoptosis (programmed cell death), which contributes to elevated reactive oxygen species (ROS) levels. The damage is further aggravated by atrophic gastric glands and reduced blood supply, which are associated with aging. These conditions lead to a weakened gastric environment. This weakening contributes to hypochlorhydria (reduced stomach acid), causing iron malabsorption, bacterial overgrowth, and vitamin B12 deficiency. This impaired environment can manifest in symptoms like delayed gastric emptying, gastro-esophageal reflux, and dyspepsia, collectively contributing to Anorexia of Aging. Additionally, the impact of external factors such as stress and NSAIDs is highlighted, indicating their role in exacerbating stomach issues in aging individuals. Create with biorender.

The aging process induces significant functional and structural changes in the stomach, distinguishing it markedly from that of a younger individual. These alterations trigger various biological responses, including disrupted communication between the nucleus and mitochondria, reduced oxygenation, increased cell apoptosis, and elevated reactive oxygen species levels [47,48,49]. A common consequence of these changes is a delay in gastric emptying, often leading to symptoms of dyspepsia [50]. Additionally, these changes can contribute to the “anorexia of aging” and postprandial hypotension [51,52,53]. Moreover, the delayed gastric emptying in older persons may also predispose them to reflux, especially if cholecystokinin secretion is heightened [51] (Figure 2).

While alterations in gastric motility are often linked to conditions such as diabetes mellitus, neurological disorders, or connective tissue diseases, they are generally not a direct result of aging alone. The combination of delayed gastric emptying, reduced mucosal thickness, compromised blood flow, and diminished mucus production significantly undermines the stomach’s protective mechanisms, predisposing older individuals to conditions such as chronic gastritis, functional dyspepsia, and gastroesophageal reflux disease (GERD). Hypochlorhydria, or reduced stomach acid production, is particularly prevalent, especially in individuals with current or past *H. pylori* infections. Reduced gastric acidity compromises nutrient absorption, particularly of iron and vitamin B12, and facilitates bacterial overgrowth in the small intestine, particularly when autoimmune atrophic gastritis and parietal cell loss further impair intrinsic factor production [51].

Moreover, chronic *H. pylori* infection, through sustained inflammatory responses, frequently leads to atrophic gastritis, resulting in compromised acid secretion, impaired nutrient absorption, and exacerbated morbidity in older individuals.

Aging also impacts the stomach’s microbial diversity. In the gastric mucosa of individuals uninfected by *H. pylori*, there is a noticeable decline in both the abundance and diversity of microbial groups, including Firmicutes (e.g., Streptococcus and Veillonella), Fusobacteria (Fusobacterium), Actinobacteria (Rothia), and Bacteroidetes (Prevotella) as people age (Shin et al., 2021). Additionally, aging is linked to broader alterations in gut microbiota composition, often resulting in a state of “dysbiosis”—characterized by reduced populations of beneficial bacterial species, including Bifidobacterium and Lactobacillus, alongside an increase in pro-inflammatory species [54]. This dysbiotic shift fosters a pro-inflammatory environment that exacerbates gastrointestinal and systemic disease risks and undermines the efficacy of *H. pylori* eradication therapies.

The interplay between *H. pylori* infection and age-associated microbiota changes establishes a context of chronic inflammation, reduced digestive efficiency, and increased susceptibility to conditions such as GERD and functional dyspepsia [28]. Immunosenescence, characterized by diminished mucosal immune responses, impaired macrophage function, and decreased immune surveillance, further exacerbates these challenges by allowing the persistence of pathogens such as *H. pylori*, ultimately raising the risk of peptic ulcers and, in severe cases, gastric carcinoma [55,56,57].

More recently, programmed cell death (PCD) and autophagy have been found as fundamental cellular processes profoundly impacted by *H. pylori* infection, contributing significantly to the pathogenesis of gastric diseases. *H. pylori* infection perturbs PCD pathways, including apoptosis, necroptosis, and pyroptosis, thereby exacerbating epithelial damage and enhancing the risk of gastric malignancy [58]. Concurrently, autophagy, a crucial mechanism for maintaining cellular homeostasis through the degradation of damaged organelles, is similarly dysregulated by *H. pylori*. The pathogen strategically modulates autophagic processes, alternately inhibiting or co-opting them, facilitating intracellular persistence and furthering inflammation and oncogenesis within the gastric mucosa [59,60] Recent pharmacological studies have identified several agents capable of modulating these dysregulated pathways. Compounds such as quercetin and L-ascorbic acid-2-glucoside (AA2G) have demonstrated efficacy in modulating intrinsic apoptotic signaling, thereby mitigating *H. pylori*-induced cellular damage [61,62]. In the context of autophagy, chloroquine has shown potential in attenuating abnormal autophagic activity by inhibiting the overexpression of autophagy-related proteins, specifically Beclin1 and LC3B-II [63,64]. These findings emphasize the dualistic nature of PCD and autophagy as both protective and pathogenic elements in *H. pylori* infection and underscore the potential of targeted therapeutic interventions aimed at these cellular pathways to alleviate *H. pylori*-associated gastric pathology.

Addressing these multifactorial challenges necessitates an integrated therapeutic approach that includes strategies to enhance immune resilience, restore microbial balance, and effectively manage chronic *H. pylori* infections, thereby mitigating the gastrointestinal complications associated with aging.

### 3.3. Challenges and Strategies in Diagnosis of H. pylori in Older Persons

Diagnostic approaches for *H. pylori* infection are broadly divided into invasive and non-invasive methods [65]. Invasive tests, such as the rapid urease test (RUT), histology, and bacterial culture, are highly accurate but require endoscopic biopsy samples, making them less suitable for routine use, particularly in older patients [66]. Non-invasive methods, including the urea breath test (UBT), serological blood test, and stool antigen test (SAT), are more commonly utilized due to their practicality, especially in older populations [67]. While international guidelines such as the Maastricht VI/Florence Consensus Report, the American College of Gastroenterology Clinical Guideline, and the Kyoto Global Consensus Report offer clear recommendations for *H. pylori* diagnosis [68], they acknowledge that the accuracy of non-invasive tests may be reduced in older persons. Given the age-related physiological changes and potential for false-negative results, diagnostic strategies must often be adjusted when managing older patients.

#### 3.3.1. Non-Invasive Tests

The diagnostic accuracy of non-invasive *H. pylori* tests varies considerably in the older population due to several physiological changes associated with aging. Among the non-invasive methods, the UBT is widely regarded as the gold standard due to its high accuracy [68]. The 13C-UBT is particularly recommended for older persons, as it uses a stable, non-radioactive isotope, making it a safer alternative to 14C-UBT [69]. Studies have demonstrated that 13C-UBT offers excellent diagnostic performance in this population, with a sensitivity of 100%, a specificity of 95.7%, and an overall accuracy of 98% [70]. 14C-UBT, while effective, shows slightly lower sensitivity (91.4%) and specificity (93.8%) [71]. However, certain physiological changes associated with aging, such as delayed gastric emptying and reduced metabolic rate, can influence UBT values in older individuals. These factors may necessitate adjusting diagnostic thresholds for patients over 60 years old [72]. Moreover, advanced gastric atrophy or intestinal metaplasia, common in older patients, can reduce *H. pylori* density in the stomach, leading to false-negative UBT results [73].

Serological testing, which detects antibodies against *H. pylori*, offers another non-invasive diagnostic option, though it is less reliable in older populations. Studies have reported a sensitivity of 74.4%, a specificity of 59%, and an overall diagnostic accuracy of 67% in older individuals [74]. One limitation is that *H. pylori* antibodies can persist for extended periods after bacterial eradication, making it difficult to differentiate between current and past infections, particularly in patients with advanced atrophic gastritis [75]. Additionally, older persons with compromised immune systems or malnutrition may have diminished antibody responses, leading to false-negative results [43]. Combining serological testing with UBT, however, may improve the overall sensitivity and specificity of *H. pylori* diagnosis in older populations [74].

The stool antigen test (SAT) is another valuable non-invasive method for detecting *H. pylori* antigens in stool samples. SAT is particularly advantageous for older individuals, as its accuracy is not affected by conditions such as atrophic gastritis, ulcers, or intestinal metaplasia [76]. SAT has shown high diagnostic accuracy (91.5%) and specificity (97.6%) in older patients, although its sensitivity is lower, at 68.7% [77]. One factor contributing to the reduced sensitivity in this age group is the higher prevalence of constipation, which can slow gastrointestinal transit and result in degradation of bacterial antigens [78]. SAT is particularly useful for patients who cannot undergo UBT, such as those with respiratory conditions or difficulties in cooperating with breath testing [79].

Tailoring diagnostic strategies to the specific physiological needs of older populations is essential for optimizing *H. pylori* detection and management in this age group. To this address, in the short-term follow-up period of 4–6 weeks after eradication treatment, it is recommended to avoid the use of antibiotics or bismuth compounds to enable accurate testing for *H. pylori*. PPIs should also be discontinued at least 14 days before testing, as these medications may suppress the bacteria without fully eradicating it, leading to misleading results. This drug-free period is crucial to differentiate true eradication from temporary suppression and avoid false negatives [14].

#### 3.3.2. Invasive Tests

Invasive diagnostic methods are commonly utilized for the detection of *H. pylori* infection in older adults, given the high prevalence of severe gastric conditions and the frequent indication for endoscopic evaluation in this population. Gastroscopy, in combination with biopsy-based diagnostic modalities, remains an effective approach for identifying gastric lesions, particularly in older patients at increased risk of gastric malignancy or presenting with alarm symptoms such as unexplained weight loss or anemia [80,81,82].

Among invasive diagnostic approaches, the rapid urease test (RUT) is widely utilized due to its rapid results and acceptable diagnostic accuracy, with sensitivity as high as 95% and specificity between 85% and 95% [81]. However, RUT sensitivity in older patients is significantly lower (57%) compared to younger individuals (75%), possibly due to the patchy distribution of *H. pylori* or interference from antisecretory drugs, which can diminish bacterial load by reducing gastric acidity, thereby affecting test accuracy. Therefore, discontinuation of antisecretory drugs prior to testing is recommended to enhance accuracy [83,84]. Furthermore, a multi-point biopsy approach, including sampling from both the antrum and corpus, is advocated to improve detection rates [84].

Histological evaluation of biopsy samples is considered the gold standard for the diagnosis of *H. pylori* infection. It offers additional diagnostic value through the assessment of morphological alterations in the gastric mucosa, such as lymphocytic infiltration, glandular atrophy, intestinal metaplasia, and mucosal erosion, which aids in evaluating the presence and severity of gastritis [85,86]. Despite its high diagnostic value, histological sensitivity may be compromised in older patients due to extensive atrophic changes within the gastric mucosa, necessitating comprehensive sampling, typically involving at least two biopsies from both the antrum and corpus [82]. The Operative Link on Gastritis Assessment (OLGA) system has been developed to stratify the severity of histological gastritis, ranging from low-risk (stage 0) to high-risk (stage IV) categories for gastric cancer. This stratification is clinically important for identifying patients at elevated risk and guiding appropriate surveillance and management strategies [85]. Furthermore, specialized staining techniques such as Giemsa and immunohistochemical staining are recommended to enhance *H. pylori* detection, particularly in cases with low bacterial density [82,87,88].

Bacterial culture of *H. pylori* is not routinely employed in clinical practice due to the challenging growth requirements of the bacterium, including its microaerophilic nature, specific nutrient requirements, and susceptibility to improper storage conditions, which contribute to its relatively low sensitivity (ranging between 53.3% and 90%) [19,89]. Nonetheless, bacterial culture plays a crucial role in antimicrobial susceptibility testing, especially in patients exhibiting resistance to standard eradication regimens. This allows for targeted treatment strategies, which are particularly important for patients in whom initial eradication attempts have failed [90].

Molecular techniques, while not routinely used as first-line diagnostic tools, exhibit high sensitivity and specificity and can be beneficial for guiding antibiotic selection in resistant cases [80]).

### 3.4. Clinical profile of H. pylori in Older Adults

Helicobacter pylori infection manifests with a diverse spectrum of clinical features, which can vary substantially depending on patient age. In younger individuals, the infection is often characterized by acute symptoms, such as epigastric pain, nausea, and vomiting [91]. In contrast, older patients typically experience more chronic conditions, including CAG, PUD, and gastric cancer (GC) [4]. It is important to note that many older patients may have acquired *H. pylori* infection at a younger age but remained asymptomatic for decades [92]. The delayed detection in older age could be linked to the progression of the infection from an asymptomatic phase to more clinically significant conditions, such as CGA or peptic ulcer disease [43]. The cumulative effect of long-term infection might contribute to the age-associated decline in gastric function and increased risk of malignancies [92]. Several studies indicate that the initial acquisition of *H. pylori* often occurs during childhood, particularly in individuals born in less hygienic conditions, which may lead to chronic, lifelong colonization [8,93,94,95,96]. Understanding this progression is essential when interpreting findings from studies involving older populations, as the natural history of the infection can influence both disease manifestation and treatment outcomes in older patients.

#### 3.4.1. Peptic Ulcer Disease

*H. pylori* infection is a primary etiological factor for PUD, chronic gastritis, and gastric mucosa-associated lymphoid tissue (MALT) lymphoma, and it is also implicated in the development of GC [27,66,69,97,98]. In older populations, the concurrent presence of *H. pylori* infection and long-term use of NSAIDs or aspirin substantially increases the risk of PUD and associated complications, such as gastrointestinal bleeding [98,99,100]. Clinical evidence suggests that eradication of *H. pylori* in older patients with PUD significantly reduces the risk of ulcer recurrence and gastrointestinal hemorrhage, particularly in the initial years following eradication. For instance, the HEAT trial demonstrated that *H. pylori* eradication significantly lowered the risk of aspirin-associated ulcer bleeding in the first 2.5 years, with a hazard ratio of 0.35 compared to the placebo group (*p* = 0.028), although this protective effect diminished with longer follow-up [101]. This finding is particularly relevant for older patients on antiplatelet therapy, such as aspirin or non-vitamin K oral anticoagulants (NOACs), who are at heightened risk of gastrointestinal bleeding due to cardiovascular comorbidities. However, proton-pump inhibitor co-therapy is often required to effectively mitigate the risks of NSAID-associated gastroduodenal damage, and ongoing monitoring may be necessary to sustain the benefits of *H. pylori* eradication in the long term.

#### 3.4.2. Chronic Atrophic Gastritis

Chronic atrophic gastritis is another prevalent manifestation of *H. pylori* infection among older adults. Prolonged infection can lead to atrophy of the gastric glands and the subsequent development of intestinal metaplasia, both of which are recognized as precancerous conditions [102]. Recent research has shown that the eradication of *H. pylori* in older individuals can significantly decrease the activity of gastritis. This intervention may also avert the progression to more advanced stages of atrophy and metaplasia, thereby the risk of developing GC [103,104]. GC, which remains one of the most common malignancies in older adults, is strongly associated with *H. pylori* infection. The bacterium is classified as a type I carcinogen and is responsible for the majority of non-cardia GCs [25,105].

#### 3.4.3. Gastroesophageal Reflux Disease

Gastroesophageal reflux disease is another significant concern in older patients with *H. pylori* infection. While some studies suggest a protective role for *H. pylori* against GERD by reducing gastric acid secretion through atrophic changes, others indicate that eradication of *H. pylori* may increase the risk of reflux symptoms and erosive esophagitis, especially in patients on long-term PPI therapy [103,106,107,108]. Consequently, a thorough evaluation is recommended before initiating eradication therapy in older GERD patients to prevent the potential exacerbation of symptoms.

#### 3.4.4. Extra-Digestive Conditions

Beyond its impact on the gastrointestinal system, *H. pylori* infection has been linked to several extra-digestive conditions in older persons patients, including iron-deficiency anemia (IDA), idiopathic thrombocytopenic purpura (ITP), and vitamin B12 deficiency [109,110]. Interestingly, in populations with a high prevalence of *H. pylori* infection, the incidence of vitamin B12 deficiency is expected to be significant. Khadim et al. found that 64% of individuals with *H. pylori* had vitamin B12 deficiency, which contributed to the early onset of chronic gastritis [111]. Carmel et al. further investigated the link between *H. pylori* infection and megaloblastic anemia in patients with impaired dietary cobalamin absorption. Their findings showed that individuals with low serum cobalamin levels had a higher prevalence of *H. pylori* infection [112]. The potential link between *H. pylori* infection and vitamin B12 deficiency becomes particularly important in this context, as the resulting deficiency is known to contribute to serious neurological conditions such as cognitive decline, dementia, psychosis, mania, and agitation, with research indicating that low serum vitamin B12 levels are associated with a 2- to 4-fold increased risk of cognitive dysfunction [113,114,115].

*H. pylori* has also been implicated in cardiovascular diseases, with some studies suggesting that chronic infection may increase the risk of coronary artery disease through its contribution to systemic inflammation and endothelial dysfunction. Additionally, there is evidence to suggest a role for *H. pylori* in the pathogenesis of metabolic syndrome, as chronic infection may contribute to insulin resistance and altered lipid metabolism. The proinflammatory effects of *H. pylori* are also believed to play a role in neurological conditions such as Alzheimer’s disease, potentially contributing to neuroinflammation and increased oxidative stress [74]. These associations underscore the far-reaching impact of *H. pylori* infection beyond the gastrointestinal tract, particularly in vulnerable old populations. Studies have shown that eradication of *H. pylori* can lead to remission of ITP and improve anemia and iron status, particularly in patients with moderate to severe IDA [109]. Furthermore, *H. pylori*-induced chronic inflammation has been associated with neurodegenerative diseases such as Alzheimer’s and Parkinson’s, although these associations require further investigation for conclusive evidence [36].

### 3.5. Challenges and Innovations in Treating H. pylori Infection Among Older Adults

The eradication of *H. pylori* infection in older adults requires a nuanced and individualized approach due to decreased gastric mucosal barrier function and the higher prevalence of chronic atrophic gastritis and intestinal metaplasia in this population [116]. The clinical benefits of *H. pylori* eradication are well established, with retrospective studies indicating a significant reduction in GC incidence following eradication, particularly in older individuals [117,118]. Moreover, eradication is especially advantageous for older adults who are frequently prescribed aspirin or NSAIDs for cardiovascular conditions or gout, as it significantly mitigates the risk of gastrointestinal complications [119]. Nevertheless, treatment regimens must be tailored to each patient’s overall physical health, comorbidities, and renal function to minimize adverse drug reactions [120,121].

#### 3.5.1. Treatment Guidelines and Challenges

Although multiple treatment guidelines are available for managing *H. pylori* infection in adults and adolescents [86,122,123,124], older adults require a thorough benefit-risk assessment to determine the appropriateness of eradication therapy [105]. Certain aspects of these guidelines can be challenging to apply directly to older adults, including the need to manage multiple comorbidities, polypharmacy, and the increased risk of drug interactions, all of which require careful adjustment of treatment protocols [121]. Studies have shown that clinicians may be hesitant to treat significantly older patients due to concerns regarding potential adverse effects [125]. This hesitation is further exacerbated by the high prevalence of antibiotic resistance among older adults, largely due to the historical overuse of antibiotics [94,126]. Additionally, beyond the aging-related factors previously mentioned, lower treatment compliance among older adults may significantly limit the effective management of *H. pylori* infections in this group [127]. A 2022 Hp-EuReg study compared eradication rates between older and younger European patients, involving 49,461 *H. pylori*-positive adults from 29 countries, of whom 14,467 were aged 60 or above. Although older patients had more concurrent medications, treatment adherence was still excellent at 97% in both groups [128]. However, the variability of data and marginal differences rendered these results clinically insignificant. Nevertheless, lower adherence remains a critical factor in optimizing treatment for older adults.

#### 3.5.2. Proton Pump Inhibitor-Based Triple Therapy

PPI-based triple therapy, comprising a PPI, clarithromycin, and either amoxicillin or metronidazole, has long been recognized as a primary therapeutic approach for *H. pylori* eradication since its endorsement in the initial Maastricht Consensus [129]. Early randomized controlled trials affirmed the effectiveness of this regimen, with eradication rates between 79.0% and 85.7% (intention-to-treat) and 82.8% to 94.0% (per protocol) in older patients over 60 years of age [36,74,130]. Notably, the administration of clarithromycin at a lower dose (250 mg twice daily) or for a shorter duration (5 days) resulted in comparable eradication rates to the higher dose (500 mg twice daily) or longer treatment duration (7 days) while also yielding fewer adverse events and reduced costs [36,130,131]. However, PPI-based triple therapy has demonstrated a diminished protective effect against NSAID-induced gastroduodenal injury compared to PPI monotherapy in older patients with *H. pylori* infection [131]. These findings highlight the need to balance the benefits of *H. pylori* eradication with the necessity for adjunctive measures to manage gastroduodenal health in older patients. Several investigations have assessed the efficacy of PPI-based triple therapy involving various antibiotics, such as amoxicillin, clarithromycin, metronidazole, or levofloxacin, across different age groups, including older and younger patients and have consistently reported comparable eradication outcomes [66,125,132,133,134,135,136,137,138,139]. These findings suggest that advanced age, per se, does not significantly diminish treatment efficacy.

However, the overall success rates vary substantially due to regional differences in antibiotic resistance, with eradication rates ranging from 40% to 95% among older patient populations. In general, the rates are higher in Japan and Singapore compared to Taiwan, underscoring notable regional disparities [66,125,132,134,135,136,137,138,139]. These variations emphasize the necessity of considering local antibiotic resistance patterns when determining appropriate treatment strategies. To this address, the Maastricht Consensus guidelines recommend PPI-based triple therapy involving clarithromycin and amoxicillin in areas where the resistance rate for clarithromycin is below 15% to 20% [14]. Conversely, in regions where the resistance rate exceeds this threshold, bismuth-containing quadruple therapy is advised as a more efficacious option, demonstrating higher eradication rates despite increased resistance [14]. A critical consideration in this context is the high prevalence of antibiotic resistance in older adults, an issue that remains underexplored. Factors such as cumulative antibiotic exposure, diminished gastric mucosal immunity, and increased bacterial colonization pressure contribute to a heightened risk of harboring resistant *H. pylori* strains [140]. Moreover, non-invasive genotypic testing, including stool-based PCR assays, has been proposed as a promising method for guiding antibiotic selection in the face of resistance; however, widespread implementation of these methods is limited by cost and availability [140]. Such individualized approaches could potentially mitigate the growing issue of resistance by tailoring treatment based on identified resistance profiles, thereby enhancing treatment success in older adults. The paucity of data specifically addressing antibiotic resistance in older adults necessitates a more personalized approach to antibiotic therapy. Tailored resistance-guided therapies, such as incorporating amoxicillin-clavulanate or utilizing molecular diagnostic tools to ascertain clarithromycin resistance, can significantly enhance eradication outcomes compared to empirical triple therapy [133,141]. This approach aligns with broader trends suggesting that properly informed empiric treatment, which considers local resistance patterns and individual risk factors, can be as effective as purely resistance-guided strategies [140].

Given these complexities, future therapeutic strategies must incorporate the assessment of individual clinical factors, including prior antibiotic use, regional resistance patterns, and patient-specific demographics, to optimize *H. pylori* therapy in older adults. As discussed in the literature, personalized treatment plans that combine susceptibility testing with clinical insights hold significant promise for addressing both antibiotic stewardship and the urgent need for more effective eradication protocols within this population.

Furthermore, vonoprazan-based regimens, which demonstrate superior gastric acid suppression compared to conventional PPIs, have emerged as promising therapeutic alternatives. Notably, a 7-day course of vonoprazan has been shown to achieve eradication outcomes that are comparable to those observed with 14-day PPI-based regimens [132,139]. Empirical evidence suggests that vonoprazan-based triple therapy—comprising vonoprazan, amoxicillin, and clarithromycin—attains eradication rates exceeding 90%, which are significantly higher than those achieved by standard PPI-based regimens [142,143]. Moreover, vonoprazan-based therapies have demonstrated considerable efficacy in both first-line and second-line settings, achieving eradication rates ranging from 91% to 95% [142,144]. These findings underscore the utility of vonoprazan not only as an initial therapeutic option but also as a potential rescue therapy in cases where previous PPI-based regimens have proven inadequate.

In addition, vonoprazan-based dual therapy—comprising vonoprazan and amoxicillin—has demonstrated efficacy comparable to that of triple therapy, while effectively eliminating the need for clarithromycin. This represents a significant advantage, particularly in regions with high clarithromycin resistance or in patients with a heightened risk of adverse drug interactions. The robust acid inhibition afforded by vonoprazan facilitates effective dual therapy, thereby mitigating the risks associated with clarithromycin, including QT interval prolongation and gastrointestinal disturbances [145,146]. These features render vonoprazan-based regimens a compelling option for optimizing *H. pylori* management, particularly in older adults who often present with multiple comorbidities and are more susceptible to adverse reactions from polypharmacy.

Although the current body of evidence supporting vonoprazan use predominantly originates from studies conducted in eastern Asia, its efficacy suggests considerable potential for application in Western populations, provided that local antibiotic resistance profiles and clinical validations are carefully considered [147]. Further research is warranted to establish the efficacy of vonoprazan-based regimens across diverse geographic regions and to explore their integration within various therapeutic frameworks, thereby potentially enhancing the global management strategies for *H. pylori* infection.

PPI-based triple therapy for *H. pylori* eradication in older adults has raised concerns about a potential increased risk of *Clostridium difficile* infection (CDI), a common healthcare-associated condition [127,148]. Although some antibiotics and PPIs are linked to a higher risk of CDI, a large retrospective study involving over 38,000 individuals, with a median age of 62 years, found no significant association between *H. pylori* treatment and an increased CDI risk [149]. Thus, even if the literature on this subject is limited, existing data suggest that the benefits of PPI-based triple therapy, particularly in preventing peptic ulcers and malignancies, generally outweigh the minimal CDI risk in this population [150,151].

Additionally, PPIs are linked to potential exacerbation of age-related skeletal changes, increasing fracture risks. This risk is thought to arise from the inhibition of bone resorption and reduced calcium absorption, demonstrated in both in vitro and in vivo studies [152,153,154,155]. Calcium absorption naturally declines with age and is influenced by dietary factors [156]. PPIs may further impair calcium absorption, although this effect was primarily observed in elderly women taking omeprazole while fasting [157].

PPIs may also induce hypergastrinemia, which stimulates parathyroid gland activity, leading to increased parathyroid hormone (PTH) secretion (up to 28%) [91], thereby enhancing bone resorption. However, Targownik et al. found no significant link between chronic PPI use and bone mineral density (BMD) reduction [158]. Four additional studies also found no BMD differences between PPI users and non-users [159]. Conversely, some evidence suggests a decline in BMD, including among adults with GERD undergoing PPI therapy [160] and reduced trabecular BMD in older adults [161], indicating a possible osteoporosis link in predisposed individuals. Anyway, it should be highlighted that the literature on fracture risk associated with PPIs is inconsistent. Some studies reported an association between long-term PPI use and increased risks of hip or other fractures, with relative risks ranging from 1.18 to 4.55 [106,158,162]. However, these studies often included various fracture types, and only a subset demonstrated a dose-response or duration effect, supporting a causal relationship [163,164].

In the end, despite the efficacy of these therapies, there remains reluctance among clinicians to prescribe eradication treatment to older patients, often due to concerns regarding potential adverse effects. However, evidence suggests that the safety profile and efficacy of PPI-based triple therapy are consistent across different age groups, including patients aged over 75 years, and similar to those observed in younger cohorts [125]. This highlights the appropriateness of PPI-based triple therapy in older individuals, provided that individual resistance data and patient-specific factors are thoroughly evaluated. Given the increasing emphasis on precision medicine, understanding the interplay of local resistance patterns, comorbidities, and other individual risk factors is pivotal to achieving optimal eradication outcomes in older patients. By integrating these considerations, clinicians can effectively mitigate the potential risks associated with treatment while maximizing the likelihood of successful eradication, ultimately enhancing the quality of care for older patients.

#### 3.5.3. Efficacy of Bismuth-Augmented PPI Triple Therapy

PPI-based triple therapy augmented with bismuth has been increasingly advocated as a primary empirical intervention for *H. pylori* eradication, particularly in response to the escalating global issue of antibiotic resistance [165]. Evidence suggests that incorporating tetracycline within this bismuth-containing quadruple regimen significantly enhances eradication rates, largely due to its low resistance profile [166]. A 14-day triple therapy combined with bismuth demonstrated eradication rates comparable to those of triple therapy with berberine in both older patients and younger cohorts, with patient age not substantially influencing treatment efficacy [141,167]. However, a study comparing a 10-day triple plus bismuth regimen with concomitant therapy revealed that the latter was superior (eradication rates of 93.5% versus 77.1%) in populations exhibiting high levels of resistance to clarithromycin and metronidazole [141]. These findings underscore the potential utility of resistance-guided tailored therapy as an effective alternative to empirical treatment [66,168]. Furthermore, bismuth, which does not develop microbial resistance, combined with a PPI-based triple regimen, confers additional benefits as a first-line treatment for *H. pylori* due to its safety profile and its role in mitigating subsequent resistance development [32,169]. Clinical studies have indicated that bismuth-containing quadruple therapy yields high eradication rates when used as a first-line treatment in older patients, with success rates surpassing 90% [169]. Nevertheless, around 28% of patients experienced mild to moderate adverse effects, especially with extended treatment durations [166,170,171]. Additionally, sequential therapy has shown greater efficacy compared to standard triple therapy, attaining higher eradication rates while producing minimal side effects [130]. Notably, a retrospective analysis of over 1200 older patients receiving *H. pylori* eradication with triple therapy indicated a low incidence of adverse events (<10%), suggesting that chronological age alone should not preclude treatment initiation [125].

#### 3.5.4. Sequential and Hybrid Therapy Strategies

Sequential and hybrid therapies have emerged as highly effective strategies for the eradication of *H. pylori*, particularly among older populations. A substantial body of evidence demonstrates that sequential therapy, which typically achieves eradication rates exceeding 90%, significantly outperforms conventional triple therapy in older patients [130,134,137]. Hybrid therapy has shown comparable efficacy to sequential therapy, further supporting its use in this demographic [136,172]. Importantly, the effectiveness of these treatment regimens does not appear to be negatively impacted by advanced age when compared to younger cohorts. The enhanced efficacy of sequential and hybrid therapies is attributed primarily to the initial administration of amoxicillin, which effectively reduces bacterial density and mitigates resistance, thereby augmenting the subsequent activity of clarithromycin [173]. Additionally, resistance to nitroimidazole does not appear to significantly compromise the efficacy of non-bismuth quadruple therapy [174,175].

Despite the promising results, regional variations in treatment efficacy have been observed. For instance, a study conducted in Korea reported only moderate efficacy of sequential therapy in older patients, although it remained superior to triple therapy, potentially due to elevated levels of antibiotic resistance in that population [176]. In China, resistance to clarithromycin or metronidazole may diminish the success rates of sequential therapy, rendering it a less favorable option for certain populations [146].

Recent investigations further emphasize the advantages of sequential treatment regimens. For example, a 10-day sequential regimen involving five days of a PPI and amoxicillin, followed by an additional five days of PPI, clarithromycin, and tinidazole, has been shown to be more effective than a 7-day course of PPI, amoxicillin, and clarithromycin [177,178]. In older adults with peptic ulcer, this 10-day sequential regimen was associated with significantly higher eradication rates compared to standard triple therapy [179].

Retrospective analyses of older patients indicate that adverse effects are generally mild (<12%) across both sequential and triple therapies, suggesting that advanced age alone should not be a contraindication for these treatment regimens [125,130].

#### 3.5.5. Efficacy and Safety of High-Dose Dual Therapy

Compared to standard bismuth-containing quadruple therapies, high-dose dual therapy has demonstrated favorable efficacy and safety profiles, particularly as a salvage option when conventional regimens fail [180,181]. The efficacy of this approach has been corroborated by several studies, including meta-analyses, which indicate that dual therapy exhibits comparable effectiveness to mainstream first-line treatment regimens, with a significantly reduced incidence of adverse side effects [182,183]. Furthermore, retrospective analyses and clinical trials have underscored that modified high-dose dual therapy, consisting of elevated doses of amoxicillin in combination with rabeprazole, is both effective and well tolerated, particularly among older patients without contraindications such as penicillin allergy or renal insufficiency [183,184,185,186,187,188]. Importantly, although the total daily dosage of amoxicillin is elevated in high-dose dual therapy, it remains within the established safety parameters. Additionally, this regimen minimizes the utilization of other antibiotics and bismuth, thereby potentially reducing treatment-related side effects and overall costs compared to bismuth-based quadruple therapy [182]. Consequently, high-dose dual therapy offers a promising approach for the eradication of *H. pylori*, especially as a salvage regimen in cases where conventional treatments have failed. Its efficacy, safety, and reduced side effects make it a compelling option, particularly for older individuals without specific contraindications. Further research is warranted to continue validating its effectiveness across diverse patient populations.

#### 3.5.6. Microbiota and Probiotic Therapy

Aging is associated with changes in gastric and intestinal microbiota, making older adults more susceptible to antibiotic-induced dysbiosis [180]. The core microflora, including Bacteroidetes, tends to decline, while the abundance of subdominant taxa such as Firmicutes, Actinobacteria, and Proteobacteria may increase [178]. Administering probiotics during or after *H. pylori* eradication therapy can help restore microecological balance and mitigate adverse gastrointestinal effects [56,179]. Recent evidence supports the adjunctive use of probiotics in *H. pylori* eradication regimens, with the goal of diminishing therapy-related side effects, enhancing patient adherence, and ultimately improving eradication success rates. A recent network meta-analysis encompassing 40 studies with 8924 participants demonstrated that probiotic supplementation significantly increased eradication rates while reducing the overall incidence of treatment-related side effects. The data further indicated that prolonged probiotic administration—before, during, and after *H. pylori* eradication therapy—was associated with the most pronounced benefits, particularly when combined with bismuth quadruple therapy [189].

Among the diverse probiotic strains investigated, *Lactobacillus* species have been particularly noted for their efficacy. Specific strains, including *Lactobacillus reuteri*, *Saccharomyces boulardii*, and *Bifidobacterium bifidum*, have demonstrated effectiveness in mitigating gastrointestinal side effects and maintaining gut microbiota stability throughout eradication therapy [9].

Two recent clinical investigations specifically evaluated *Lactobacillus reuteri* in combination with PPIs as a potential alternative to antibiotics. In a Romanian cohort of 23 patients with functional dyspepsia, this regimen achieved an eradication rate of 65%, whereas the outcomes were less favorable in an Italian cohort, where only 3 out of 24 patients (12%) experienced successful eradication [169,190].

Moreover, additional studies have identified other beneficial probiotic formulations, including a combination of *Bifidobacterium animalis lactis* BB12 and *Enterococcus faecium* L3, which exhibited modest but positive effects as adjunctive therapies in *H. pylori* eradication. These findings suggest that probiotic therapy may play a pivotal role in enhancing both therapeutic efficacy and patient tolerability, particularly by ameliorating antibiotic-induced dysbiosis and supporting the maintenance of a balanced gut microbiota during and after treatment [169,191].

#### 3.5.7. Economic Considerations

Economic analyses have demonstrated that *H. pylori* eradication is cost-effective, particularly for reducing the incidence of GC in high-risk populations [1]. In Japan, eradication therapy administered between 2013 and 2019 led to a marked decrease in both the incidence and mortality of gastric cancer among patients aged 20–80 years, with the most substantial health and economic benefits observed in those around 60 years of age [192]. These findings underscore the value of *H. pylori* eradication not only in improving patient outcomes but also in alleviating the economic burden on healthcare systems, particularly in high-risk older populations.

#### 3.5.8. Towards Effective *H. pylori* Management in the Aging Population

Older patients are particularly susceptible to antibiotic resistance due to extensive, long-term use of antibiotics, such as fluoroquinolones for respiratory or urinary tract infections, which has led to increased resistance to drugs like metronidazole and levofloxacin [171]. This highlights the importance of conducting drug susceptibility testing to ensure effective and individualized therapy for *H. pylori* Additionally, age-related declines in hepatic and renal function elevate the risk of adverse drug reactions due to impaired drug metabolism, and these risks are heightened by interactions between commonly used medications, such as PPIs and cardiovascular agents [193,194]. These challenges necessitate individualized risk–benefit assessments for eradication therapy, especially in older adults with comorbidities or frequent NSAID use [86]. In this population, therapeutic strategies must be carefully adapted to consider their unique physiological vulnerabilities. The balance between treatment benefits and risks must be evaluated individually, factoring in comorbidities, potential drug–drug interactions, life expectancy, and quality of life. Effective eradication therapy in older adults should involve simplified combination regimens to promote adherence and minimize adverse effects. Close monitoring of drug–drug interactions between antibiotics and other medications is also critical for optimizing outcomes (Tomita et al., 2019). Reducing the complexity of medication schedules can significantly improve adherence, ultimately leading to better clinical outcomes. For instance, simplified triple therapies or shorter-duration regimens can minimize the adverse effects associated with extended antibiotic use, which is particularly important for those with renal or hepatic impairment. Additionally, emerging therapies, such as vonoprazan-based regimens and high-dose dual therapy, offer simplified treatment pathways with promising efficacy and safety profiles for older patients [195]. Additionally, regimens that include fewer drugs or shorter treatment durations may also help minimize the risk of adverse drug reactions and drug–drug interactions, thereby improving the overall safety profile of *H. pylori* eradication in this vulnerable population. For certain patients, particularly those over 80 years of age or with significant organ dysfunction, the risks associated with eradication therapy may outweigh the benefits. In these cases, alternative approaches, such as probiotics or natural substances with anti-*H. pylori* properties may offer complementary or substitute solutions [196,197,198]. Integrating these non-antibiotic strategies could provide viable options for patients who cannot tolerate conventional treatments.

Considering the increased prevalence of gastric cancer in the aging population, a comprehensive diagnostic and therapeutic approach for *H. pylori* is crucial. This includes tailoring eradication regimens based on thorough assessments of functional, cognitive, nutritional, and social factors, as well as accounting for comorbidities and concurrent treatments. A multidisciplinary team approach ensures that all aspects of a patient’s health are integrated into treatment planning, optimizing patient outcomes [42,43]. Recent evidence supports the involvement of a multidisciplinary team in formulating individualized treatment plans, thereby maximizing the benefit-risk ratio of eradication regimens [199,200,201]. Geriatricians and gastroenterologists collaboratively assess each patient’s suitability for eradication, considering not only the clinical burden of *H. pylori* but also potential complications related to the patient’s existing comorbidities and medication profile. Such an approach allows for a careful selection of therapeutic protocols, including alternative regimens that minimize antibiotic use to mitigate dysbiosis and other age-associated side effects, thus improving tolerability and treatment success rates [16,34].

## 4. Conclusions

This review provides a comprehensive examination of the complex relationship between *H. pylori* infection and the aging process, underscoring the increased vulnerability of older populations to gastrointestinal complications and the significant challenges associated with managing these infections. The aging process is characterized by distinct pathophysiological changes, such as immunosenescence, multimorbidity, and an elevated risk of adverse pharmacological interactions, which collectively exacerbate the difficulties in diagnosing and treating *H. pylori*. Despite these multifaceted challenges, there is an urgent need to develop individualized treatment strategies that prioritize both therapeutic efficacy and the overall quality of life for elderly patients. Emerging therapeutic modalities, such as vonoprazan-based regimens, high-dose dual therapies, and hybrid or sequential treatment protocols, offer promising pathways to address these treatment complexities.

Future research should focus on refining diagnostic methodologies specifically adapted to the physiological characteristics of older adults, addressing factors such as reduced diagnostic accuracy due to age-related gastric alterations and polypharmacy. Investigating novel therapeutic regimens that demonstrate both efficacy and tolerability in older populations is crucial, particularly given the increasing prevalence of antibiotic resistance, the need to optimize proton pump inhibitor (PPI) utilization, and the imperative to minimize adverse effects. Moreover, exploring the potential of adjunctive interventions—including probiotics, natural bioactive compounds, and precision medicine approaches—may yield innovative strategies to mitigate the deleterious effects of *H. pylori* infection in older adults.

A multidisciplinary approach is indispensable to deliver comprehensive care, necessitating the collaboration of gastroenterologists, geriatricians, and other healthcare professionals to achieve a holistic and individualized management plan for this vulnerable cohort. By advancing our understanding of the interplay between aging and *H. pylori* pathogenesis, we can enhance therapeutic outcomes, diminish the burden of gastric diseases in the elderly, and improve overall health and quality of life. Expanding research efforts in these domains will be pivotal in addressing the unique needs of older adults and optimizing the clinical management of *H. pylori*-associated conditions in an aging global population.

## Data Availability

No new data were created or analyzed in this study.
